# Pre-treatment anemia in head and neck cancer: risk factors, subtypes, and survival outcomes

**DOI:** 10.3389/fonc.2025.1577901

**Published:** 2025-06-25

**Authors:** Cornelius H. L. Kürten, Vinithagowry Sivakumar, Sebastian Waßenberg, Marie Carolin Schleupner, Valentin Funk, Elena Lazzarini, Sara Aksu, Maja Guberina, Thomas Gauler, Martin Stuschke, Stefan Mattheis, Stephan Lang, Timon Hussain

**Affiliations:** ^1^ Department of Otorhinolaryngology, Head and Neck Surgery, University Hospital Essen, University of Duisburg-Essen, Essen, Germany; ^2^ punkt05 Statistikberatung, Düsseldorf, Germany; ^3^ Department of Radiation Oncology, University Hospital Essen, University of Duisburg-Essen, Essen, Germany; ^4^ Klinikum rechts der Isar, Munich Technical University, Department of Otorhinolaryngology, München, Germany

**Keywords:** cancer associated anemia, head and neck cancer, squamous cell carcinoma, HPV+ oropharyngeal cancer, iron deficiency, macrocytic anemia, vitamin B12, folate

## Abstract

**Introduction:**

Anemia in head and neck cancer (HNC) may be due to additive, overlapping or competing causes. Here we aim to investigate the prevalence of pre-therapeutic anemia, associated risk factors, subtypes, and impact on survival among HNC patients, including the clinically relevant subgroups of HPV-associated HNC.

**Materials and methods:**

A retrospective chart review of HNC patients diagnosed between 2010 and 2020 identified a study cohort of 921 patients. Every patient was subject to pre-therapeutic laboratory testing which was correlated with clinical data and oncologic outcomes.

**Results:**

Pre-therapeutic anemia was present in 18.1% of patients with advanced age, low BMI, systemic inflammation, and kidney dysfunction being significant risk factors for anemia. For oropharyngeal cancers, p16+ status was associated with lower anemia prevalence and offset the impact of smoking history on anemia risk when compared to all HNC patients. 19.2% of patients were hypochromic-microcytic and 4.2% were hyperchromic-macrocytic, indicative of iron or vitamin B12/folate deficiency, respectively. Even mild anemia (11- 12.9 g/d) was associated with a survival disadvantage compared to non-anemic patients (64% vs. 85% overall survival, <0.001).

**Conclusion:**

Anemia is a significant negative survival predictor in HNC patients, with severity affecting prognosis. A relevant subgroup of patients had potentially reversible anemia subtypes, early identification and treatment of which may improve outcomes.

## Introduction

Most risk factors affecting cancer patients’ treatment response and disease prognosis are not modifiable at the time of diagnosis. For head and neck cancer (HNC) patients, these include a history of smoking, alcohol consumption and poor dental hygiene (1). This holds true for patients with non-HPV-related HNC, as well as HPV-associated HNC patients, albeit to a lesser degree (2). Anemia, defined by the WHO as hemoglobin levels <13 g/dl for men or < 12 g/dl for women, is a common condition in the general population, but is increased in cancer patients due to disease pathophysiology as well as treatment effects such as bone marrow toxicity or intraoperative blood loss, a phenomenon termed cancer-associated anemia (3). Interestingly, there are reports from non-HNC cohorts such as in patients receiving chemotherapy for non-myeloid malignancies (4) as well as colon cancer patients undergoing surgery with curative intent (5) showing improved outcomes if the comorbid cancer associated anemia is treated.

Even though the prognostic impact of anemia in HNC patients has been previously explored (6–14), details of its pathophysiology and risk factors in the context of HNC remains unclear. Importantly, in this patient population, the occurrence of anemia may have additive, overlapping or competing reasons. Dysphagia and malnutrition as well as chronic alcohol consumption, which are common in HNC patients, are known risk factors for mineral or vitamin deficiencies, such as iron, vitamin B12 or folate (15), which in turn cause different subtypes of anemia. Also, HNC patients potentially loose blood due to chronic bleeding from the primary tumor. Further, inflammatory, and other regulatory cytokines, that are secreted as part of the tumor-host-interaction, may interfere with erythropoiesis (16).

In this study, we retrospectively identified a cohort of close to 1000 consecutive patients treated at our institution between 2010 and 2020. We then applied descriptive statistics, subgroup comparisons, and multivariable regression models to explore clinical and pathological factors that put patients at risk for anemia and analyzed their impact on survival. Importantly, we also differentiated anemia subtypes, i.e. hyperchromic-macrocytic, normochromic-normocytic, hypochromic-microcytic, which may point to the underlying pathophysiology and allow for treatment to improve patient outcomes.

## Materials and methods

### Patients

**Figure 1:**
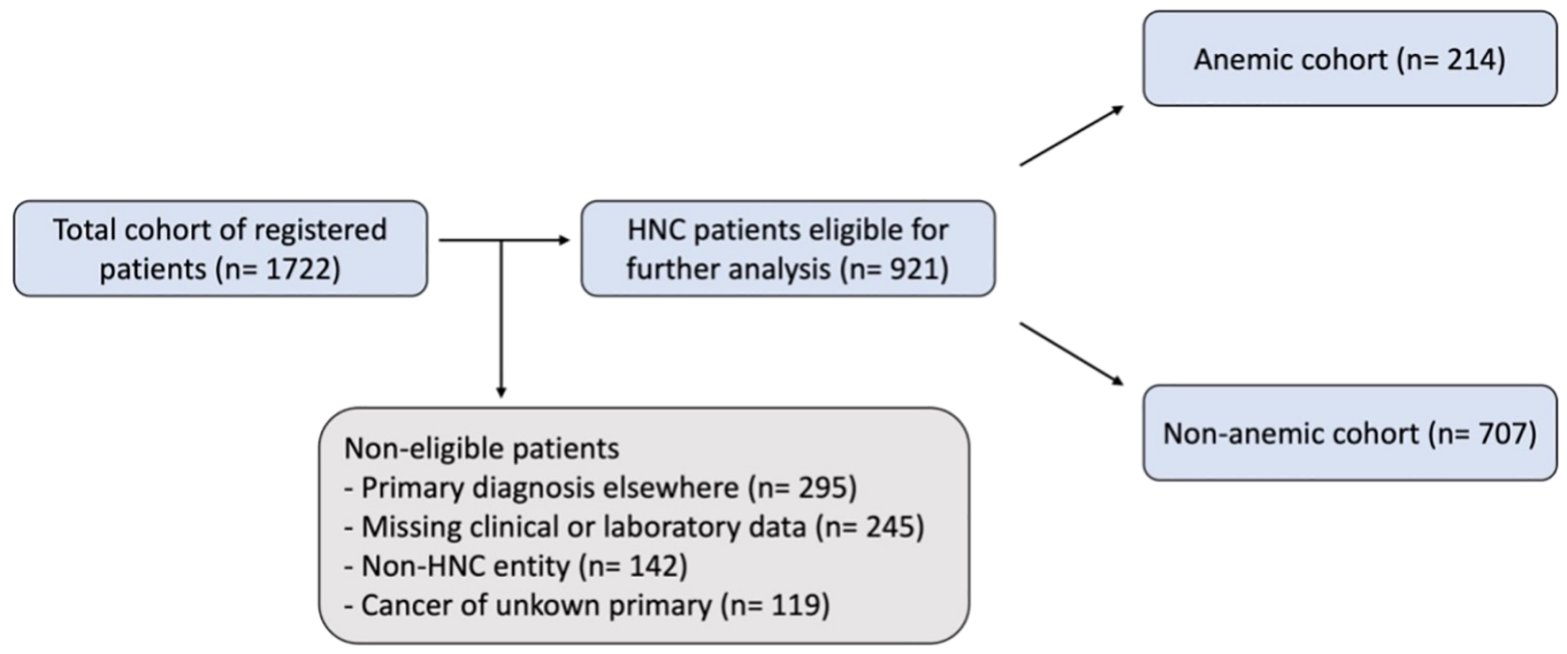
Flow chart of patient selection. An initial total cohort of registered patients was retrieved from the central certification and quality management system, this was then reviewed using digitalized paper and electronic records leading to the study cohort of HNC patients eligible for further analysis. WHO/CTCAE criteria were applied to classify patients as anemic or non-anemic.

We performed a retrospective chart review for 1722 consecutive patients that were registered at our cancer center’s central certification and quality management system between 2010 and 2020. A consecutive list of all patients was systematically created using electronic and digitized paper records. Inclusion criteria were histologically proven cancer of the head and neck and age > 18. 801 patients were subsequently excluded from the analysis: 295 patients’ primary diagnosis was established at another hospital system, 245 patients were excluded due to missing relevant data at diagnosis, 119 patients initially presented with a cancer of unknown primary, and 142 patients had non-HNC malignancies, such as skin cancer, lymphoma, or skull base malignancy (**Figure 1**). The relevant clinical, demographic, and pathological data was extracted, including age, gender, tumor location, clinical TNM staging, primary treatment modality, laboratory results and date of recurrence or death. Patients for which these data were not available were excluded from the analysis. All data other than survival outcomes were collected at diagnosis.

For all patients, pre-treatment laboratory testing was part of the workup prior to tumor panendoscopy, or parotidectomy in the case of parotid tumors. Anemia was defined as a hemoglobin level < 13 mg/dl (Grade 1 11.0 - 12.9 mg/dl; Grade 2 8.0 – 10.9 md/dl; Grade 3 < 8.0 mg/dl) in men and < 12 mg/dl (Grade 1 11.0 - 11.9 mg/dl; Grade 2 8.0 - 10.9 md/dl; Grade 3 < 8 mg/dl) in women using a combination of the World Health Organization (WHO) criteria and US Food and Drug Administration’s Common Terminology Criteria for Adverse Events (CTCAE) as previously described (3). Ranges for MCV and MCH were defined as 85–98 fl and 28–34 pg. Anemia subtypes were defined using these standard hematologic thresholds; hypochromic-microcytic anemia was defined as both MCH and MCV below the reference range; hyperchromic-macrocytic anemia as both parameters above the reference range; normochromic-normocytic anemia as both parameters within range; and mixed subtypes were assigned when only one of the two indices deviated. Patients were staged according to the current edition of the AJCC guidelines (AJCC Staging Manual 7^th^ Edition up to 2017, AJCC Staging Manual 8^th^ Edition starting in 2018). Body mass index (BMI, self-reported weight in kilograms per height in meters squared) was calculated at diagnosis and classified into underweight (<18.5 kg/m^2^), normal weight (18.5 - 24.9 kg/m^2^), overweight (25 - 29.9 kg/m^2^) and obese (≥ 30 kg/m^2^). Kidney function was grouped by GFR according to the CKD stages of KDIGO 2012 criteria into the categories stage 1 + 2 GFR ≥ 60, stage 3 GFR 30-59, stage 4 GFR 15–29 and stage 5 GFR <15 (all ml/min/1.73m^2^) (17).

### Survival data

We performed an automated update of survival data via a population registry query. For this purpose, the Institute of Medical Informatics, Biometry and Epidemiology (IMIBE Essen) queries the data at the electronic registration portal of the authorities (German “*Einwohnermeldeamt*”), with a cutoff date on April 27, 2023.

### Statistical analysis

Statistical analysis was performed using the SPSS Software (Version 29 for MAC). Numerical variables such as age were reported as mean values with standard deviation, while categorical variables were presented as absolute numbers and percentages. For comparisons between categorical variables, the chi-square test was applied. Continuous variables following a normal distribution were compared between two groups using the unpaired t-test. When comparing more than two independent groups, the Kruskal-Wallis test was used to detect differences in the distribution of continuous variables. Bonferroni correction was applied where applicable to account for multiple comparisons.

To evaluate predictors of pretherapeutic anemia, univariable and multivariable binary logistic regression analyses were performed. Covariates included relevant demographic and clinical parameters, and results were reported as odds ratios (OR) with 95% confidence intervals.

Survival analysis was performed using Kaplan-Meier curves to illustrate overall survival, and the log-rank test was used to compare survival distributions between groups. By convention, a p-value of <0.05 was considered significant. This study was performed in line with the principles of the Declaration of Helsinki. The study protocol was reviewed and approved by the ethics committee of the University of Essen, Germany (internal reference code: 21-9893-BO).

## Results

### Patient characteristics and prevalence of anemia

**Table 1** shows all patient’ characteristics. 18.1% of patients were anemic (167/921), 13% (124/921) had mild, 4% (39/921) moderate and 0.4% (4/921) severe anemia according to WHO/CTCAE criteria. Patients’ mean age was about five years higher in the anemic compared to the non-anemic group (67.7 years vs. 62.9 years, p < 0.001). Females had a lower risk for anemia than males (16% vs. 26%, p = 0.008). Former alcohol addiction was associated with higher risk of anemia (15% vs. 8%, p = 0.047), while active alcohol consumption as well as active or former tobacco consumption were not associated with significantly higher anemia prevalence. BMI as a surrogate for nutritional status showed an increased number of anemic patients being underweight (14% vs. 4%), while obese patients showed a lower prevalence of anemia (23% vs. 10%, p < 0.001). Anemia prevalence did not differ between patients with histologically proven SCC from non-SCC HNC. Hypopharyngeal cancer was associated with higher anemia risk compared to other HNC locations, while p16+ oropharyngeal cancer was associated with lower anemia risk (15% vs. 8% and 2% vs. 6% respectively, p= 0.028). Stage I HNC was associated with lower anemia prevalence (23% vs. 15%, p < 0.001), while patients in stage IVc were more likely to be anemic (9% vs. 1%, p < 0.001). A significantly higher percentage of patients with anemia had elevated CRP levels compared to the non-anemic patients (71% vs. 39%, p < 0.001). There was no association with INR as a sign of liver dysfunction or warfarin therapy. Anemic patients were more likely to have kidney dysfunction (KDIGO stages III, IV and IV) than non-anemic patients (p < 0.001). As a primary treatment modality, anemic patients more often than non-anemic patients received radiochemotherapy (39% vs. 30%, p < 0.001) and thus were less likely to undergo surgery (47% vs. 68%, p < 0.001).

**Table 1:** Patients characteristics and comparison between anemic and non-anemic patients.

Total cohort etc	Total cohort n = 921	Patients without anemia n = 754 (81.9%)	Patients with anemia n = 167 (18.1%)	p-value
Age (years)	63.8 (11.4)	62.9 (10.7)	67.7 (12.6)	**<0.001**
Gender Male Female	n= 921 76% (699) 24% (222)	n= 754 74% (559) 26% (195)	n= 167 84% (140) 16% (27)	**0.008** **+** **+**
Body Mass Index	n= 487	n= 415	n= 72	**<0.001**
Underweight Normal weight Overweight Obese	5% (27) 42% (202) 32% (157) 21% (101)	4% (17) 40% (168) 33% (136) 23% (94)	14% (10) 47% (34) 29% (21) 10% (7)	**+** – – **+**
Alcohol consumption	n= 707	n= 597	n= 110	**0.047**
None Abuse S/p abuse	70% (491) 21% (150) 9% (66)	71% (422) 21% (126) 8% (49)	63% (69) 22% (24) 15% (17)	– – **+**
Smoking	n= 730	n= 613	n= 117	0.234
Never Active S/p abuse	26% (189) 56% (409) 18% (132)	27% (166) 55% (339) 18% (108)	20% (23) 60% (70) 20% (24)	– – –
Histological subtype	n= 915	n= 749	n= 166	0.344
SCC Non- SCC	96% (875) 4% (40)	95% (714) 5% (35)	97% (161) 3% (5)	– –
Localization	n= 921	n= 754	n= 167	**0.028**
Nose/paranasal sinuses Nasopharynx Oropharynx; AJCC 7 Oropharynx; AJCC 8 p16- Oropharynx; AJCC 8 p16+ Hypopharynx Larynx Oral cavity Salivary glands	5% (44) 4% (38) 24% (223) 5% (46) 5% (47) 9% (89) 32% (290) 12% (106) 4% (38)	5% (38) 5% (34) 23% (175) 5% (36) 6% (44) 8% (63) 32% (242) 12% (90) 4% (32)	4% (6) 2% (4) 29% (48) 6% (10) 2% (3) 15% (26) 29% (48) 10% (16) 3% (6)	– – – – **+** **+** – – –
Stage	n= 897	n= 735	n= 162	**<0.001**
I II III IVa IVb IVc	21% (190) 16% (146) 17% (148) 41% (364) 3% (31) 2% (18)	23% (166) 16% (121) 17% (126) 40% (296) 3% (22) 1% (4)	15% (24) 15% (25) 14% (22) 42% (68) 5% (9) 9% (14)	**+** – – – – **+**
INR	n= 912	n= 748	n= 164	0.735
≤ 1,5 > 1,5	98% (898) 2% (14)	98% (737) 2% (11)	98% (161) 2% (3)	- -
KIDGO criteria	n= 913	n= 748	n= 165	**<0.001**
1 + 2: eGFR 60 to >90 3: eGFR 30-59 4: eGFR 15-29 5: eGFR <15	83% (761) 15% (141) 1% (6) 1% (5)	87% (651) 13% (96) 0% (0) 0% (1)	67% (110) 27% (45) 4% (6) 2% (4)	**+** **+** **+** **+**
CRP (mg/dL)	n= 899	n= 734	n= 165	**<0.001**
< 0,5 ≥ 0,5	55% (499) 45% (400)	61% (451) 39% (283)	29% (48) 71% (117)	**+** **+**
Primary Therapy	n= 910	n= 748	n= 162	**<0.001**
Surgery Radiochemotherapy Palliation Declined	65% (587) 31% (283) 4% (36) 0% (4)	68% (511) 30% (219) 2% (15) 0% (3)	47% (76) 39% (64) 13% (21) 1% (1)	**+** **+** **+** –

Data of metric variables in mean (standard deviation) or median and interquartile range (Q1-Q3), data of categorical variables in % (n); significant results are highlighted in bold, s/p = status post, SCC, squamous cell cancer; AJCC, American Joint Committee on Cancer; INR, International Normalized Ratio; KIDGO, Kidney Disease Improving Global Outcomes; eGFR, Estimated Glomerular Filtration Rate; CRP, c-reactive protein.Significant results are highlighted in bold

### Subclassification by erythrocyte indices into anemia subtypes

**Figure 2:**
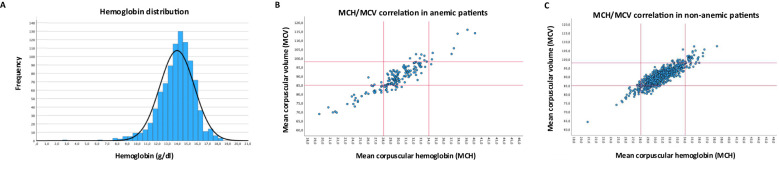
Hemoglobin concentration and erythrocyte indices. **(A)** Distribution of hemoglobin concentration across the whole cohort. **(B)** Correlation of MCH and MCV in anemic patients **(C)** Correlation of MCH and MCV in non-anemic patients, red lines in B/C indicate cut-off for normal upper and lower limit.

**Figure 2A** shows the hemoglobin concentration across the total cohort (mean: 13.96 mg/dl). Subclassification of anemic patients by erythrocyte indices (**Figure 2B**; **Supplementary Table S1**) shows 63% to be normochromic-normocytic, 19% patients to be hypochromic-microcytic, and 4% to be hyperchromic-macrocytic, while 14% of patients were classified outside of this framework (7% normochromic-microcytic, 4% normochromic-macrocytic, 1% hyperchromic-normocytic, 2% hypochromic-normocytic). In the non-anemic cohort, 79% of patients were normochromic-normocytic, 5% hypochromic-microcytic and 4% hyperchromic-macrocytic (**Figure 2C**; **Supplementary Table S1**).

### Risk factors of anemia and its subtypes

To minimize the impact of confounding variables, we performed a multivariable regression analysis of anemia risk in our cohort (**Table 2**). Here, advanced age had a small, but significant effect (OR: 1.06; p < 0.001), as did active or former smoking status (OR: 1.97; p = 0.019) and decreased kidney function (GFR < 60; OR: 2.38; p = 0.002). Gender, alcohol consumption and tumor stage did not influence patients’ anemia risk.

**Table 2:** Multivariable regression of anemia risk factors in HNC.

Total cohort etc	Odds ratio [95% CI]	p- value
Age (years)	1.06 (1.03- 1.08)	**<0.001**
Gender		
male female	reference 0.57 (0.32- 1.02)	- 0.060
Alcohol consumption		
no abuse + s/p abuse	reference 1.35 (0.21- 1.34)	- 0.211
Nicotine		
never active + s/p abuse	reference 1.97 (1.12- 3.45)	- **0.019**
Kidney function		
eGFR ≥ 60 eGFR < 60	reference 2.38 (1.39- 4.07)	- **0.002**
Tumor stage		
I/II III/IV	reference 1.40 (0.88- 2.21)	- 0.152

eGFR, Estimated Glomerular Filtration Rate; s/p, status post.Significant results are highlighted in bold.

Further, we aimed to identify certain risk factors associated with anemia subtypes (**Supplementary Table S2**): hypochromic-microcytic anemia was associated with younger age and kidney dysfunction was more prevalent in normochromic-normocytic anemia. Other characteristics such as gender, BMI, alcohol consumption, smoking, cancer stage and localization and CRP levels were not associated with anemia subtype.

### Impact of anemia and anemia subtype on overall survival

**Figure 3:**
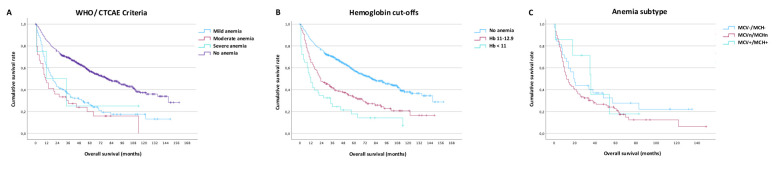
Impact of anemia on survival. **(A)** Overall survival by WHO/CTCAE criteria for mild, moderate, and severe anemia, **(B)** Overall survival using internal optimized cut-offs ≥ 13, 11-12.9 and <11, **(C)** Overall survival depending on classic anemia subtype (hypochromic-microcytic, normochromic-normocytic, hyperchromic-macrocytic).

Overall survival was analyzed using Kaplan-Meier plots. Anemia according to WHO/CTCAE criteria was associated with worse overall survival (**Figure 3A**; **Supplementary Table S3**). We optimized the cut-offs to identify clinically applicable values for mild (11- 12.9 g/d) and moderate-severe (< 11 g/d) anemia which showed good separation regarding overall patient survival (**Figure 3B**; **Supplementary Table S3**). Even mild anemia was associated with a pronounced survival disadvantage when using gender-independent hemoglobin levels of 11- 12.9 g/d (1-year: 64%, 5-year: 33%) compared to non-anemic patients (1-year: 85%, 5-year: 57%, <0.001). Anemia subtype does not significantly influence overall survival (**Figure 3C**; **Supplementary Table S3**).

### Subgroup analysis of oropharyngeal cancer patients (OPSCC)

**Figure 4:**
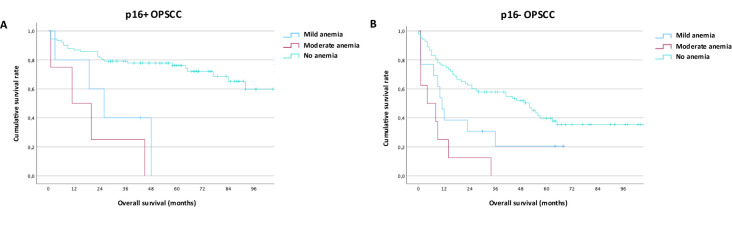
Impact of anemia on outcomes in p16-positive and p16-negative oropharyngeal cancer. **(A)** Overall survival in p16-positive oropharyngeal cancer by WHO/CTCAE criteria for mild and moderate anemia **(B)** Overall survival in p16-negative oropharyngeal cancer by WHO/CTCAE criteria for mild and moderate Anemia.

Out of 328 OPSCC patients in the total cohort, 104 were p16-positive, 109 patients were p16-negative, and for 115 patients the p16-status was unknown. As expected, compared to p16-negative patients, p16+ OPSCC had a better overall survival both using the AJCC8 Staging system as well as looking at p16-positive patients independent of the year of diagnosis (data not shown). Among patients with known p16-status, anemia was associated with worse survival in both the p16-positive (**Figure 4A**) and the p16-negative (**Figure 4B**; **Supplementary Table S3**) patient subgroup. In a separate regression analysis of only oropharyngeal cancers (**Table 3**), the addition of p16-status had a pronounced protective effect in positive patients again anemia risk (OR: 0.21, p = 0.031) and offset the abovementioned effect of smoking, while age (OR 1,07, p = 0,023) and kidney function (OR: 5,63, p = 0,009) remained relevant.

**Table 3:** Univariable/multivariable regression of anemia risk factors in oropharyngeal cancer.

Total cohort etc	Odds Ratio (95% CI)	p-value
Univariable Regression		
p16- status		
negative positive	reference 0.40 (0.17–0.92)	**0.032**
Multivariable Regression		
Age (years)	1.07 (1.01–1.13)	**0.023**
Gender		
male female	reference 0.53 (0.14–1.95)	0.336
Alcohol consumption		
no abuse + s/p abuse	reference 1.44 (0.48- 4.28)	0.516
Nicotine		
never active + s/p abuse	reference 1.54 (0.25- 0.34)	0.642
Kidney function		
GFR ≥ 60 GFR < 60	reference 5.63 (1.53- 20.59)	**0.009**
p16- status		
negative positive	reference 0.21 (0.05–0.86)	**0.031**
Tumor stage		
I/II III/IV	reference 0.46 (0.16- 1.36)	0.161

eGFR, Estimated Glomerular Filtration Rate; s/p, status post.Significant results are highlighted in bold.

## Discussion

Anemia is common in cancer patients and has been shown to be prognostically relevant across multiple cancer types (18, 19). In HNC patients treated with primary radiation with or without chemotherapy the influence of anemia on treatment response has been studied extensively in retrospective (6) and prospective (7) analyses. A pathophysiological relationship between intra-tumoral hypoxia, a known risk factor for low treatment response, and anemia, which causes low blood oxygenation (20), has been proposed. Hereby, if blood oxygenation is low due to anemia, ionizing radiation might not be able to induce enough radical oxygen species, which are a main component of its indirect anti-tumor effect. Similarly, chemotherapy effectiveness may be impaired in anemic patients due to reduced tissue perfusion, altered drug pharmacokinetics, and impaired bone marrow recovery.

In patients treated with upfront surgery, the negative prognostic effect of anemia was observed in laryngeal cancer (8), oral cavity cancer (9), oropharyngeal cancer (10) and mixed cohorts across different HNC locations (11–13). Even in recurrent-metastatic HNC patients, hemoglobin levels above the median are associated with improved survival (14). Our data confirms pretherapeutic anemia to be a significant negative predictor for survival. Of note, even mild anemia with hemoglobin levels of 11.0- 12.9 g/d was associated with a significant survival disadvantage. Importantly, we demonstrate a dose-dependent relationship between the severity of anemia and overall survival: lower hemoglobin levels are associated with worse survival.

Given that HPV-associated HNC is recognized as a distinct clinical and biological entity, we performed a subgroup analysis for oropharyngeal HNC patients with known p16-status. Here, we confirmed previous findings demonstrating that p16-negagtive oropharyngeal HNC is associated with more severe anemia as compared to p16-positive cancers (10). Nevertheless, anemia confers a decreased survival probability in both p16-positive and p16-negative HNC. This is an important finding as prognostic factors to identify poor outcomes in p16-positive oropharyngeal cancer are lacking (21). We also found an increased prevalence of anemia in patients with hypopharyngeal HNC; this may result from occult blood loss or a more pronounced effect of hypopharyngeal tumors on patients’ diet.

Cancer-related anemia is a state of impaired erythropoiesis which is a complex physiological process involving multiple organs, including the liver, gut, kidney, and bone marrow to maintain homeostasis (22). Underlying causes can lead to decreased production, i.e. ineffective erythropoiesis, or increased consumption via destruction or loss/bleeding (3). In our patients, all laboratory results were collected prior to therapy and were therefore not treatment-related (23).

To identify potential risk factors and further decipher the underlying pathology, we leveraged our large patient cohort to perform a subclassification of anemia in HNC by erythrocyte indices. Most anemic patients in our collective were normochromic-normocytic. Despite the strong association of HNC with lifestyle factors, only a minority of patients had megaloblastic anemia which is associated with folate or vitamin B12 deficiency and is common in patients with increased alcohol consumption. Interestingly, a large subgroup of anemic patients presented with hypochromic-microcytic erythrocytes, indicative of iron deficiency. Even in the non-anemic patient cohort, several patients presented with altered erythrocyte indices suggesting an increased risk for future anemia. Changes to the erythrocyte indices are a late occurrence in anemia development due to the long lifespan of erythrocytes (24), hence diagnosis of deficiencies may oftentimes be delayed when relying on a basic blood panel.

An issue complicating anemia pathophysiology is differentiating overlapping causes of anemia, particularly in cases of iron-deficiency vs. inflammation-related anemia. Inflammation induced hypoferremia limits iron availability for microbes and causes bone marrow reprogramming. Decreased erythropoiesis in favor of leukocyte production strengthens host defense but may in turn lead to mild to moderate anemia (16). Laboratory testing for ferritin, transferrin, transferrin receptor and CRP can help distinguish between inflammation-induced anemia and iron-deficiency related anemia but rarely allows for a definitive diagnosis in intermediate cases (25). In our cohort CRP levels≥ 0.5 mg/dL as a marker of systemic inflammation were significantly more common in the anemic than the non-anemic patient population. When comparing anemia subtypes, increased CRP levels tended to be more common in the normochromic-normocytic than in the hypochromic-microcytic or hyperchromic-macrocytic patients, without reaching statistical significance. Chronic kidney disease was identified in this study as a risk factor for anemia in HNC patients consistently across analysis and subgroups with normochromic-normocytic anemia cancer patients showing significantly increased prevalence of kidney dysfunction. The interplay this kidney disease and other risk factors of anemia are complex (26) and indeed, given the data presented here, a large subgroup of HNC patients likely suffers from a multifactorial anemia caused by chronic inflammation, chronic kidney disease and (relative) iron deficiency.

Treatment of cancer-associated anemia remains an area of debate. The two most obvious options for treating anemia, i.e. blood transfusions and erythropoiesis-stimulating agents (ESA), come with a range of potential side effects. The addition of ESA to curative HNC treatment regimens in particular has been controversially discussed with clinical trials showing detrimental (27) or non-superior (28) survival outcomes in ESA-treated to non-ESA-treated patients. Worse survival outcomes in the ESA groups might be due to erythropoietin receptor (29) or EPHB4 (30) expression on HNC cells leading to cancer cell stimulation and disease progression. An updated Cochrane review including over 20,000 patients across tumor types confirmed a decreased need for blood transfusions in the ESA group, but also showed a marked increase in thromboembolic events, hypertension, thrombocytopenia/hemorrhage, and mortality (31). Current ASCO/ASH clinical practice guidelines therefore advise against using ESA in the curative setting (32). Similarly, blood transfusions, though effective in raising hemoglobin levels and alleviating anemia symptoms, were associated with worse survival outcomes in two recent retrospective analyses of HNC patients (11, 33). Other blood transfusions risks include infection, allergic reaction, alloimmunization, and iron overload (34).

Our abovementioned findings may provide guidance to identify alternative and more specific treatment options for HNC patients with pretherapeutic anemia. Both hypochromic-microcytic and hyperchromic-macrocytic anemia are potentially treatable with supplementation. Even pre-anemic patients with functional iron deficiency may benefit from supplementation (3). A randomized trial of intravenous versus oral iron sulfate in patients with non-myeloid malignancies showed a marked increase in hemoglobin in both groups with i.v. iron being better tolerated (35). In another randomized, placebo-controlled study in patients with non-myeloid malignancies, 50.8% of patients maintained hemoglobin levels within 0.5 g/dL of baseline after i.v. iron supplementation compared to 35.3% of patients in the placebo group (4). Further, in a study on 266 solid gastrointestinal cancer patients with pretherapeutic anemia, patients with intravenous iron therapy had markedly reduced transfusion rates 9.9% vs. 38.7% as well as hospital stay length during their respective therapeutic regimen (8.4 vs. 10.9 days) (5). These studies emphasize the importance of identifying and treating patients with potentially reversible anemia early and they suggest supplementation strategies which may be applicable in select HNC patient groups.

To support the clinical translation of our findings, we propose a structured diagnostic and management checklist for pretherapeutic anemia in head and neck cancer patients, which is based on our findings and existing hematologic principles (**Supplementary Table S4**). This tool outlines a stepwise approach from initial screening to possible interventions. While exploratory in nature, this framework may serve as a foundation for prospective validation in future studies.

Limitations of this study include the retrospective and single institution character, which limits generalizability to other contexts and could be overcome by multicenter validations We generated our study cohort based on our institutions central quality management system resulting in a large proportion of patients being excluded initially because they were not diagnosed at our department, had non-HNC entities, or missing data. This might introduce a selection or information bias if these exclusions disproportionately affected certain subgroups.

A further limitation of this study is the absence of extended laboratory parameters (ferritin, transferrin, vitamin B12, or folate), which are not part of standard pre-operative testing. These would be necessary to classify anemia subtypes with more certainty and guide specific treatment recommendations. While erythrocyte indices allow to infer likely etiologies, this approach is limited. Future prospective studies should incorporate comprehensive laboratory workups to validate these findings.

This study was designed to evaluate anemia at the time of diagnosis, prior to the initiation of cancer therapy. While longitudinal monitoring of hemoglobin levels during treatment was beyond the scope of this analysis, future prospective studies should explore anemia dynamics and their potential impact on outcomes. Even though we were able to collect surrogate markers for overall health such as age, liver function and kidney function, we did not have access to systematically collected data on comorbidities such as the ECOG Performance Status Scale or the Charlson Comorbidity Index which may be confounders associated with cancer, anemia and survival outcomes.

Some results, particularly in smaller subgroups such as OPSCC, were accompanied by wider confidence intervals, which not only limit statistical precision but also raise questions about the clinical relevance of these findings. This underscores the broader point that statistical significance alone is not sufficient but that effect sizes and their clinical implications must be considered in context. A further potential limitation of the multivariable regression approach is that we did not perform a formal analysis for multicollinearity among independent variables. While the variables included were selected based on clinical plausibility and statistical significance in univariable analysis, underlying correlations cannot be excluded. These correlations could lead to unstable coefficient estimates or mask true associations, thus affecting the validity and interpretability of the regression results. Future prospective studies should incorporate formal multicollinearity diagnostics, such as calculating variance inflation factors, to strengthen the interpretation of individual effect estimates.

In conclusion, our findings demonstrate that cancer-associated anemia is prevalent among HNC patients at initial diagnosis; likely causes include malnutrition, renal insufficiency, and systemic inflammation. Of note, we were able to demonstrate a dose-dependent relationship between anemia severity and patients’ overall survival. In patients with p16-positive oropharyngeal cancer, anemia could serve as a biomarker to identify patients at risk of poorer outcomes. Crucially, our data suggest that anemia represents a potentially modifiable risk factor in selected HNC patients who may profit from iron or vitamin supplementation. Anemia at diagnosis might further serve as a practical triaging tool to identify patients who could benefit from nutritional evaluation, hematologic work-up, or prehabilitation before initiating therapy.

## Data Availability

The original contributions presented in the study are included in the article/**Supplementary Material**. Further inquiries can be directed to the corresponding author.
