# Supplementary material for: Pre-treatment anemia in head and neck cancer: risk factors, subtypes, and survival outcomes
Source: Front Oncol. 2025 Jun 25;15:1577901. doi: 10.3389/fonc.2025.1577901 (PMC12237912; doi:10.3389/fonc.2025.1577901)
Supplement: Supplementary file 1 [file Table1.docx]

**Supplemental Table S1: Anemia subtype prevalence by erythrocyte index**

|  | **Total cohort** | **Patients without anemia** | **Patients with anemia** | **p-value** |
| --- | --- | --- | --- | --- |
| Microcytic/ hypochromic  Microcytic/ normochromic  Microcytic/ hyperchromic  Normocytic/ hypochromic  Normocytic/ normochromic  Normocytic/ hyperchromic  Macrocytic/ hypochromic  Macrocytic / normochromic  Macrocytic/ hyperchromic | n= 921  8% (71)  7% (67)  0% (0)  1% (12)  76% (696)  1% (13)  0% (0)  3% (24)  4% (38) | n= 754  5% (39)  7% (56)  0% (0)  1% (9)  79% (590)  2% (12)  0% (0)  2% (17)  4% (31) | n= 167  19% (32)  7% (11)  0% (0)  2% (3)  63% (106)  1% (1)  0% (0)  4% (7)  4% (7) | **<0.001**  **+**  –  –  –  **+**  –  –  –  – |

**Supplemental Table S2: Risk factors for common anemia subtypes**

|  | **all n = 167** | **MCV-/MCH- n = 32** | **MCVn/MCHn n = 105** | **MCV+/MCH+ n = 7** | **p-value** |
| --- | --- | --- | --- | --- | --- |
| **Subtypes** |  |  |  |  |  |
| Age (years) | 67.73 (12.61) | 61.66 (16.04) | 70.0 (10.60) | 68.99 (11.03) | **0.002**  +;– ; – |
| Gender  male  female | n= 144  85% (122)  15% (22) | n= 32  78% (25)  22% (7) | n= 105  87% (91)  13% (14) | n= 7  86% (6)  14% (1) | 0.500  –  – |
| Body Mass Index  underweight  normal weight  overweight  obese | n= 62  15% (9)  48% (30)  27% (17)  10% (6) | n= 13  0% (0)  31% (4)  46% (6)  23% (3) | n= 43  19% (8)  53% (23)  23% (10)  5% (2) | n= 6  17% (1)  49% (3)  17% (1)  17% (1) | 0.148  –  –  –  – |
| Alcohol consumption  no  abuse  s/p abuse | n= 97  62% (60)  23% (22)  15% (15) | n= 21  67% (14)  14% (3)  19% (4) | n= 71  61% (43)  25% (18)  14% (10) | n= 5  60% (3)  20% (1)  20% (1) | 0.858  –  –  – |
| Nicotine  never  active  s/p abuse | n= 102  19% (19)  61% (62)  21% (21) | n= 23  30% (7)  48% (11)  22% (5) | n= 74  13,5% (10)  65% (48)  22% (16) | n= 5  40% (2)  60% (3)  0% (0) | 0.206  –  –  – |
| Localization  Nose/paranasal sinuses  Nasopharynx  Oropharynx; AJCC 7  Oropharynx; AJCC 8 p16-  Oropharynx; AJCC 8p16+  Hypopharynx  Larynx  Oral cavity  Salivary glands | n= 144  4% (6)  3% (4)  28% (40)  7% (10)  1% (2)  17% (24)  28% (40)  9% (13)  3% (5) | n= 32  6% (2)  9% (3)  25% (8)  6% (2)  3% (1)  16% (5)  19% (6)  13% (4)  3% (1) | n= 105  4% (4)  1% (1)  29% (31)  7% (7)  1% (1)  18% (19)  28% (30)  8% (8)  4% (4) | n= 7  0% (0)  0% (0)  14% (1)  14% (1)  0% (0)  0% (0)  58% (4)  14% (1)  0% (0) | 0.531  –  –  –  –  –  –  –  –  – |
| Stages  I  II  III  IVa  IVb  IVc | n= 139  14% (19)  14% (20)  14% (19)  43% (60)  6% (8)  9% (13) | n= 30  17% (5)  3% (1)  23% (7)  37% (11)  7% (2)  13% (4) | n= 102  12% (13)  16% (16)  10% (10)  47% (48)  6% (6)  9% (9) | n= 7  14% (1)  43% (3)  29% (2)  14% (1)  0% (0)  0% (0) | 0.139  –  –  –  –  –  – |
| KDIGO (eGFR)  1+2  3  4  5 | n= 142  68% (96)  27% (38)  3% (5)  2% (3) | n= 32  78% (25)  16% (5)  6% (2)  0% (0) | n= 103  67% (69)  28% (29)  3% (3)  2% (2) | n= 7  29% (2)  57% (4)  0% (0)  14% (1) | **0.048**  –; **+**; –  –  –  – |
| CRP (mg/dL) | 1.6 (0.0- 3.7) | 1,15 (0.0–3.1) | 1.6 (0.0–3.9) | 2,4 (0.0–3.2) | 1.000  – |
| CRP (mg/dL)  < 0.5  ≥ 0.5 | n= 142  30% (43)  70% (99) | n= 32  38% (12)  62% (20) | n= 103  27% (28)  73% (75) | n= 7  43% (3)  57% (4) | 0.410  –  – |
| RDW | 45.9  (43.6- 51.4) | 44.4  (42.1–50.5) | 45.9  (43.9–49.0) | 59.7  (52.6–66.2) | 1.000; **0.004; 0.007**  –; **+; +** |

Data of metric variables in mean (standard deviation) or median and interquartile range (Q1-Q3), data of categorical variables in % (n); results of pairwise comparisons of column percentages are marked with - and +, where + indicates a significant result; significant results are highlighted in bold. s/p = status post, SCC = squamous cell cancer, AJCC = American Joint Committee on Cancer, INR = International Normalized Ratio, KIDGO = Kidney Disease Improving Global Outcomes, eGFR = Estimated Glomerular Filtration Rate, CRP = c-reactive protein, RDW = Red Cell Distribution Width

**Supplemental Table S3: Probability of survival**

|  | **12 months** | **24 months** | **36 months** | **48 months** | **60 months** | **p-value**  **[95%- CI]** |
| --- | --- | --- | --- | --- | --- | --- |
| **Overall survival** |  |  |  |  |  |  |
| Anemia Grade^1^  None  Grade 1 (mild)  Grade 2 (moderate)  Grade 3 (severe) | 85%  60%  46%  50% | 75%  43%  36%  50% | 69%  36%  27%  25% | 64%  31%  24%  - | 57%  27%  20%  - | **<0.001**  55- 71  14- 24  6- 16  0- 44 |
| Hemoglobin (g/dl)  Hb ≥ 13  Hb 11- 12.9  Hb < 11 | 85%  64%  47% | 76%  48%  35% | 70%  41%  24% | 64%  37%  21% | 57%  33%  18% | **<0.001**  66- 94  15- 31  7-17 |
| Subtype of anemia  MCV/MCH low  MCV/MCH normal  MCV/MCH high | 66%  50%  86% | 44%  36%  71% | 40%  30%  36% | 32%  27%  36% | 28%  23%  18% | 0.278  12- 26  8- 16  16- 56 |
| p16- positive OPSCC^1^  None  Grade 1 (mild)  Grade 2 (moderate) | 87%  80%  50% | 81%  60%  25% | 79%  40%  - | 78%  0%  0% | 76%  -  - | **<0.001**  –  11- 41  0- 30 |
| p16- negative OPSCC^1^  None  Grade 1 (mild)  Grade 2 (moderate) | 76%  39%  25% | 63%  31%  13% | 58%  21%  0% | 52%  -  - | 40%  -  - | **<0.001**  31- 73  7- 15  0- 14 |

^1^WHO/ CTCAE criteria mild anemia (11- 12.9 in men, 11- 11.9 in women), moderate (8- 10.9), severe (<8), OPSCC = Oropharyngeal squamous cell carcinoma

**Supplemental Table S4: Checklist for diagnostic evaluation and supportive management of pretherapeutic anemia**

| **Step 1: Routine Screening** |
| --- |
| Measure hemoglobin, MCV and MCH in all HNC patients at diagnosis as part of initial oncologic workup |
| **Step 2: Identify Anemia** |
| Use WHO/CTCAE criteria to define anemia |
| **Step 3: Classify Anemia by Erythrocyte Indices** |
| Hypochromic-microcytic (suggestive of iron deficiency) |
| Normochromic-normocytic (suggestive of chronic disease or renal insufficiency) |
| Hyperchromic-macrocytic (suggestive of vitamin B12/folate deficiency) |
| **Step 3: Second-Line Work-Up Depending on Subtype** |
| Hypochromic-microcytic: Check iron, ferritin, transferrin saturation, soluble transferrin receptor (sTfR) , CRP |
| Hyperchromic-macrocytic: Check vitamin B12 and folate levels |
| Normochromic-normocytic: Consider screening for renal function |
| **Step 4: Consider Pre-Treatment Intervention** |
| For reversible subtypes (iron, vitamin b12 or folate deficiency), consider supplementation |
| Consider hematology referral in complex or unexplained cases |
| **Step 5: Document and Monitor** |
| Longitudinal hemoglobin monitoring during treatment in future protocols to assess anemia dynamics |
